# Herpes Zoster

**Published:** 1886-01

**Authors:** Henry Wile

**Affiliations:** Atlanta, Ga.; Lecturer on Diseases of the Skin in the Atlanta Medical College


### Clinic Reports.

### HERPES ZOSTER.

BY HENRY WILE, M. D., LECTURER ON DISEASES OF THE SKIN IN THE ATLANTA MEDICAL COLLEGE.

Reported for THE ATLANTA MEDICAL AND SURGICAL JOURNAL.

You see here, gentlemen, a case of herpes zoster, commonly known as shingles, in which the process is at its height, with the cutaneous eruption presenting all the characteristics of the disease and being accompanied by those marked subjective symptoms. The patient experienced an acute neuralgic pain in the left side six days ago, and three days later the eruption appeared. As to variety, it is *abdomino-femoralis*, and as to cause, in this case it is attributable to sudden exposure. The patient was at the skating rink one night, and after indulging in the exercise of roller-skating, in which the hip joints and muscles are especially called into action, he was thrown into a state of profuse perspiration. In this state he went out into the cool night air, and a few hours later experienced sharp pain in the lumbar region.

The pathological process consists of an inflammation in the course of the cutaneous nerves involving the neurolemma, or sheath of the nerve, and secondarily, the structures of the skin.

The inflammation runs an acute course in ten to fourteen days, healing spontaneously. Where the process has been intense or subject to external irritation ulcerations of the skin may supervene that are, as a rule, obstinate to treatment. The eruption is usually unilateral, and seldom attacks an individual more than once. As to diagnosis, the disease may be confounded with vesicular eczema, and as characteristic differential features I especially call your attention to the glistening vesicles, the distinct grouping of the lesions, their tendency to remain intact throughout the entire course of the disease, not rupturing as in eczema, and the prodromic subjective symptoms, especially acute neuralgic pain. The pain may become so intense as to keep the patient awake at night, so that it is often a distressing symptom. Furthermore, I would call your attention to the occurrence of the lesions in streaks following the course of the cutaneous nerves, also to their sharp limitation by the median line.

As the disease is self-limited and disappears spontaneously, the treatment is palliative, although it is claimed by some that by the application of electricity in the form of the constant galvanic current, the process can be aborted. There is no doubt but that the constant current will often ease the pain and give relief. Five to fifteen cells are required, according to the strength of the battery, and it may be used twice daily, ten to twenty minutes at a time. Locally soothing astringent lotions, dusting powders and emollient ointments may be employed. A simple ointment of vaseline or oxide of zinc spread on muslin and sprinkled with powdered opium, renewed twice daily, according to the method of Hebra, in many cases proves efficient. Then again, the fluid extract of grindelia robusta, eight drachms to eight ounces of water, is also useful. This latter will be prescribed in this case, directing the patient to bathe the parts three or four times a day with the lotion, and following it by an application of some dusting powder, such as equal parts of oxide of zinc and powdered starch. The patient states that the pain keeps him awake at night. He will therefore be ordered to take a powder containing one-fourth grain of morphine at night, and to repeat the dose if necessary.

